# Epidemiology, clinical correlates, and management of focal periphyseal oedema (FOPE) in adolescent knees: retrospective analysis of one thousand, two hundred and one knees

**DOI:** 10.1007/s00264-026-06818-y

**Published:** 2026-05-04

**Authors:** Benjamin Petry, Ismail Khosravi, Jürgen Wansch, Michael Ban, Balazs Sztankay-Böck, Isabella Dornauer, Rainer Biedermann, Daniel Junker

**Affiliations:** 1 Department of Orthopaedics and Traumatology, Innsbruck Medical University, Innsbruck, Austria; 2 Department of Radiology, Community Hospital Hall in Tirol, Hall in Tirol, Austria; 3 Orthopädie Ban, Innsbruck, Austria; 4 Department of Surgery, Paracelsus Medical University Salzburg, Salzburg, Austria; 5 CTi, Innsbruck, Austria

**Keywords:** FOPE, Focal periphyseal edema, Adolescent knee pain, MRI, Magnetic resonance imaging

## Abstract

**Background:**

Focal periphyseal edema (FOPE) is a characteristic magnetic resonance imaging (MRI) finding in adolescent knee joints with open physes. Although described as a benign and likely self-limiting condition, large population-based studies are scarce. The aim of this study was to retrospectively analyse the incidence and the epidemiological background of this MRI finding, and to correlate FOPE lesions with clinical symptoms and treatment.

**Methods:**

This retrospective case–control study included a total of 1201 knee MRI scans from 897 patients, performed between 2007–2016 at our institution in patients aged ten to 16 years. MRIs were screened for the presence of FOPE and other pathologies. FOPE severity was categorised as mild, moderate and severe, based on lesion size and MRI appearance. Patients’ medical records were screened for symptoms, trauma history and treatment.

**Results:**

Out of the 1201 MRI scans, 97 FOPE lesions (10.8%) in 93 patients (10.4%) were identified. FOPE was significantly more frequent in girls than in boys (*p* = 0.007). FOPE was the main MRI finding in 53.6% of patients. Mild FOPE was most common (53.6%). Severe FOPE occurred only when FOPE was the main pathology (*p* = 0.005). Most FOPE patients (74.2%) were treated conservatively.

**Conclusion:**

FOPE lesions are a common MRI finding in adolescents presenting with knee pain. They tend to be more severe when occurring as the sole finding and have a significantly higher incidence in females. In this large clinical cohort, FOPE was mainly managed conservatively, supporting its interpretation as a benign, self-limiting differential diagnosis of adolescent knee pain.

## Introduction

Knee pain is a frequent complaint in children and adolescents and one of the main reasons for referral to paediatric orthopaedic services. The differential diagnosis includes multiple conditions ranging from overuse syndromes and osteochondrosis to intra-articular pathology, referred pain, and trauma-related lesions. Magnetic resonance imaging (MRI) has become the most sensitive modality to detect periarticular and intra-articular pathology when the clinical examination and radiographs are inconclusive [1, 2].

Focal periphyseal oedema (FOPE) was first described by Zbojniewicz and Laor as a focal bone marrow oedema pattern centered on the closing physis of the adolescent knee [3]. Subsequent research in the form of multiple case reports and small series have confirmed FOPE as a physeal-centered oedema with typical MRI morphology and a presumed relation to physiological physeal fusion and local mechanical stress [3–6]. Reported cohorts are small (typically 1–4 patients), and mostly focus on imaging appearance and short-term clinical course rather than incidence or management [7–10].

Existing literature shows that FOPE functions as a painful indicator of physiological physeal closure in some adolescents, but it also appears as an unexpected finding in unrelated medical conditions or even in asymptomatic knees [3, 6]. The greater trochanter serves as an example of a FOPE lesion described beyond the knee joint [10]. However, robust epidemiological data and treatment patterns in larger populations are lacking.

Our study primarily investigated the imaging incidence of FOPE, aiming to quantify its occurrence in a large consecutive cohort of adolescent knee MRIs and characterise the epidemiology of FOPE with respect to age, sex, trauma history, lesion localisation, and severity. Furthermore, our aim was to evaluate the correlation of FOPE with clinical presentation and treatment, and perform formal statistical testing for selected associations.

## Materials and methods

### Study design and setting

This retrospective case–control study was conducted at our university hospital and approved by the local ethics committee (AN2017-0010 369/4.16). All patient and imaging data were anonymised and numerically coded prior to analysis.

We identified all knee MRIs performed between 1 January 2007 and 31 December 2016 in patients aged ten to 16 years at our institution. Patients were included if the physis of the examined knee was still open. Those with closed physes or outside the age range were excluded. In total, 1201 knee MRIs of 897 individual patients met the inclusion criteria.

MRI examinations were performed using institutional musculoskeletal protocols including turbo-inversion recovery magnitude (TIRM) and proton density fat-suppressed (PD-FS) sequences as well as conventional T1- and T2-weighted turbo spin-echo sequences in three planes. Periphyseal regions with increased signal on fat-suppressed sequences and corresponding decreased signal on T1-weighted images centred around the physis were classified as suspicious for FOPE.

All MRIs were examined for the presence of FOPE lesions, which were subsequently classified according to their severity by two experienced musculoskeletal radiologists.

For each FOPE lesion, localisation (distal femur, proximal tibia, proximal fibula; isolated or combined) and signal intensity were recorded. Based on the statistical occurrence, the lesions were categorised into three types depending on lesion severity.

**Fig. 1:**
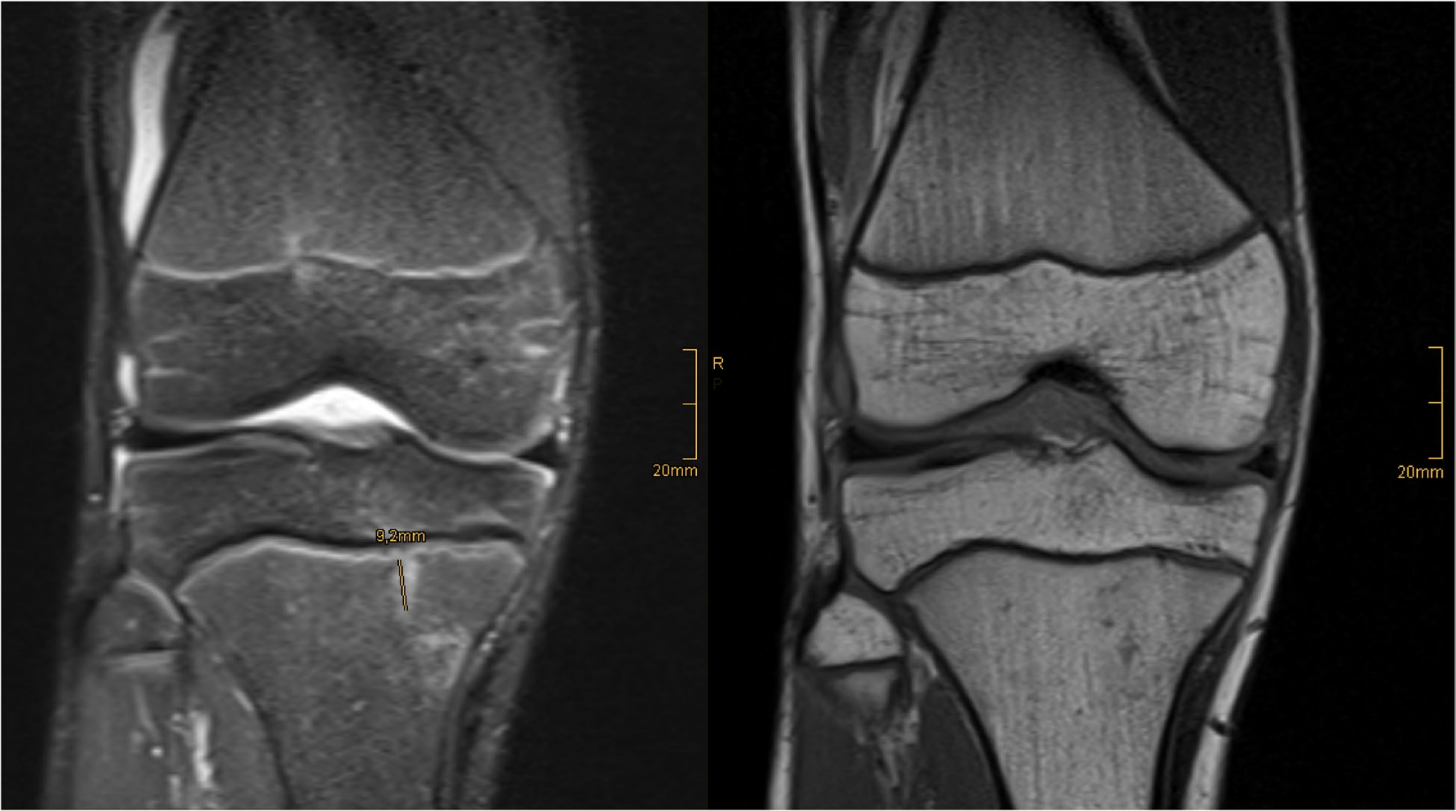
Mild: diameter < 1 cm, mild signal intensity in STIR, no or only minimal signal loss in T1
- **Moderate**: diameter 1–2 cm, moderate STIR/PD-FS hyperintensity, moderate T1 signal loss.
- **Severe**: diameter >2 cm, marked hyperintensity on STIR/PD-FS, pronounced hypointensity on T1 (Fig. 2).

This severity was graded semi-quantitatively on coronal images as:

- **Mild**: lesion diameter <1 cm, mild hyperintensity on STIR/PD-FS, no or minimal signal loss on T1 (Fig. 1).

**Fig. 2:**
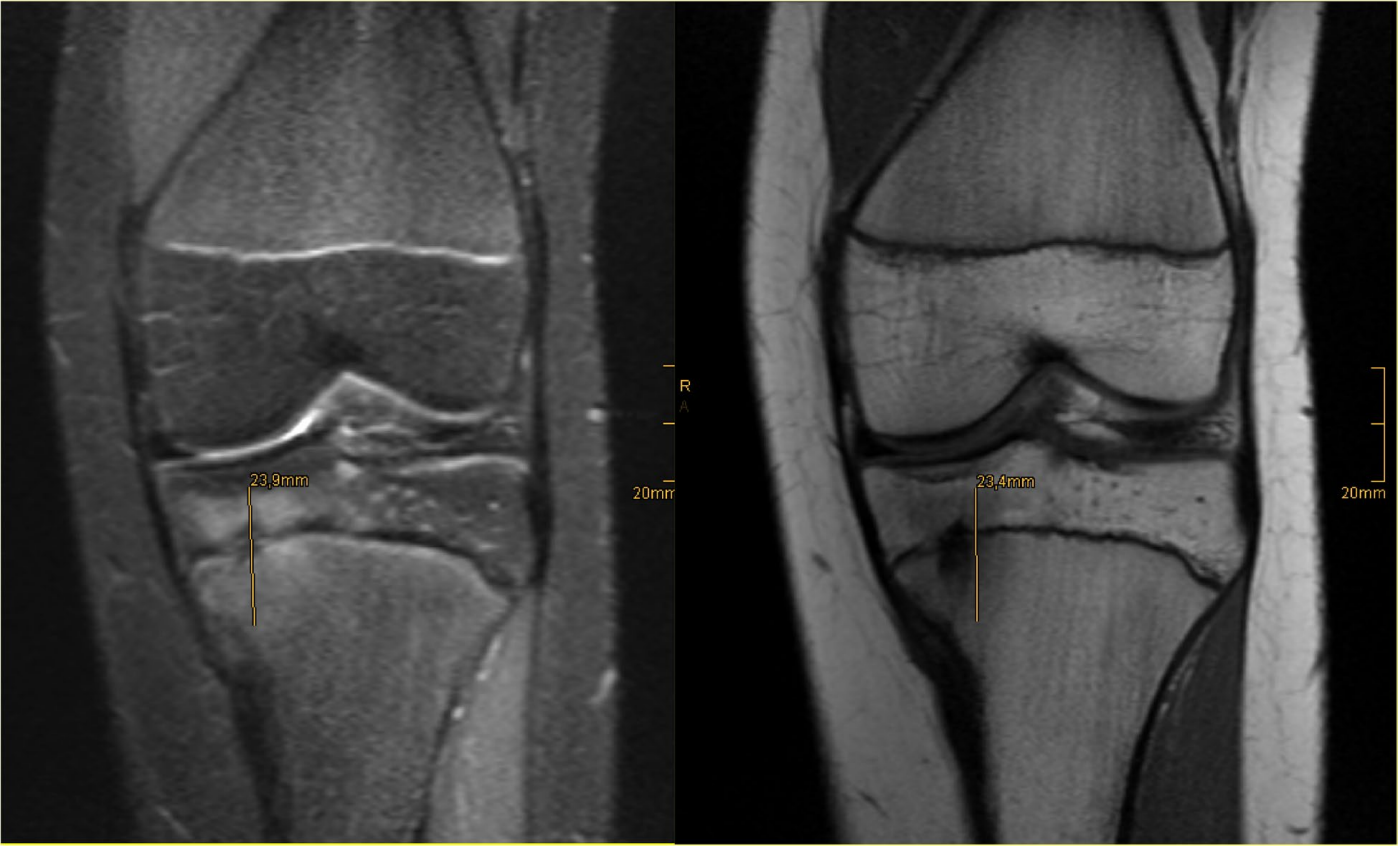
Severe: > 2 cm diameter, high signal Intensity in STIR, markedly low signal in T1

If FOPE was the only relevant pathology on MRI, it was classified as the primary pathology. In cases where other MRI pathologies were present (e.g. ligament rupture, meniscal tear, benign tumour, overuse-related changes), FOPE was categorised as a secondary finding.

Clinical documentation was obtained from the hospital information system. For FOPE-positive patients we recorded age at MRI, sex, reason for referral, and presence or absence of trauma history, and knee pain at presentation. Treatment was grouped into six categories: physical rest, physiotherapy, analgesia, rheumatological work-up, surgical procedures (arthroscopy, ligament reconstruction, etc.). Duration of treatment and number of documented follow-up visits were also recorded.

### Statistical analysis

Continuous variables were summarised as mean and standard deviation (SD) or median and interquartile range, as appropriate. Categorical variables were expressed as absolute and relative frequencies. Between-group comparisons were performed using chi-square tests for categorical variables (e.g. sex-specific FOPE incidence, severity vs main/secondary pathology) and Welch’s t-test for age comparisons. A p-value < 0.05 was considered statistically significant.

Sex-specific FOPE incidence was calculated using the total number of MRI patients by sex as denominator (426 females, 471 males). Statistical analysis was performed using IBM SPSS Statistics (Version 26.0, IBM Corp., Armonk, NY, USA).

## Results

The study included 897 patients (426 female, 471 male; median age 14.2 years, range 11.1–15.9) who underwent 1201 knee MRIs, identifying 97 FOPE lesions (10.8%) among 93 patients (10.4%). In four patients FOPE was bilateral (Table 1).

**Table 1:** Demographics of patients with FOPE

Patients Gender (f/m) Median age [y]	93 57/36 14.2 [11.1–15.9]
FOPE lesions	97
- Unilateral - Bilateral	93 4
Trauma	
- History of trauma - No history of trauma	43 (44.3) 54 (55.7)
Localisation	
- Femur - Tibia - Femur and tibia - Femur, tibia and fibula - Fibula and tibia	8 (8.3) 16 (16.5) 65 (67) 7 (7.2) 1 (1.0)
Severity	
- Mild - Moderate - Severe	52 (53.6) 35 (36.1) 10 (10.3)
*FOPE* focal periphyseal edema	

The occurrence of FOPE lesions was more frequent in female than male patients (57 of 426 females (13.4%) versus 36 of 471 males (7.6%)). The sex-specific incidence difference was statistically significant (χ^2^ = 7.3, *p *= 0.007), indicating that girls in this age range are more likely to exhibit FOPE on knee MRI.

The majority of FOPE lesions were age-dependent and emerged around the expected time of physeal closure. No FOPE zones were observed in patients aged ten to 11 years. The data revealed that the highest occurrence rate appeared in females aged 14–15 years and males aged 15–16 years, consistent with established sex-specific differences in skeletal maturation (Table 2).

**Table 2:** FOPE lesions: Gender and age distribution (%)

	FEMALE	MALE
FOPE LESIONS	58	39
AGE [y]		
- 10–11 - 11–12 - 12–13 - 13–14 - 14–15 - 15–16	0 4 (6.9) 11 (19.0) 14 (24.1) 19 (32.8) 10 (17.2)	0 6 (15.4) 2 (5.1) 6 (15.4) 9 (23,1) 16 (41.0)
*FOPE* focal periphyseal edema		

FOPE predominantly involved more than one bone: in 65 of 97 lesions (67%) the distal femur and proximal tibia were affected simultaneously; seven of 97 lesions involved femur, tibia, and fibula. Isolated femoral or tibial lesions were less common.

Regarding lesion severity, 52 of 97 (53.6%) lesions were graded as mild, 35 of 97 (36.1%) as moderate, and ten of 97 (10.3%) as severe. No correlation between lesion severity and gender could be observed (p≈0.15), the mean age also did not show a significant correlation between severity groups.

FOPE was considered the main MRI pathology in 52 of 97 lesions (53.6%) and a secondary finding in 45 of 97 (46.4%). Baseline age did not differ significantly between patients with FOPE as main pathology and those with FOPE as secondary finding (mean 13.97 vs 14.11 years; p≈0.60).

However, FOPE severity was strongly associated with whether FOPE represented the main or a secondary pathology.

- **Main pathology (*n* = 52):** mild 44.2%, moderate 36.5%, severe 19.2%.
- **Secondary finding (*n* = 45):** mild 64.4%, moderate 35.6%, severe 0%.

The statistical analysis showed a significant difference in distribution (χ^2^ = 10.5, df = 2, *p* = 0.005), indicating that severe FOPE was observed only when FOPE served as the main pathology and never as a secondary finding. The findings support the idea that larger FOPE sizes correspond to higher clinical significance.

In 54 FOPE lesions, knee pain was not associated with trauma, although 43 cases had documented a trauma history. The main MRI finding of FOPE appeared in 33 of 54 (61.1%) atraumatic cases and 19 of 43 (44.2%) traumatic cases. The difference did not show a statistical significance (χ^2^ = 2.12, *p *= 0.15), suggesting that FOPE can represent the principal pathology both with and without previous trauma.

Knee pain at presentation was documented in 88 of 93 (90.7%) FOPE-positive patients. The main diagnosis never included FOPE in the original reports from referring orthopaedic surgeons and radiologists, although the MR images clearly demonstrated the pathology, which shows that medical professionals lacked knowledge about this condition during the time of the study. FOPE was first described in 2011, our data collection spanned from 2007 to 2016.

Physicians identified other primary conditions in patients who did not have FOPE as their main diagnosis. These diagnoses included ligament injuries (35.6%), bony injuries (8.9%), meniscal tears (8.9%), benign tumours (22.2%), chronic overuse syndromes (17.8%), synovitis (2.2%), and Baker’s cyst (4.4%).

Information regarding treatment was available for 93 FOPE-positive patients. Overall, 69 of 93 (74.2%) were treated conservatively:

- Physiotherapy: 36/69 (52.2%).
- Analgesic medication: 24/69 (34.8%).
- Rheumatological work-up: 4/69 (5.8%).
- Physical rest or activity modification: 35/69 (50.7%).
- Splinting or casting: 19/69 (27.5%).

Nine patients (9.7%) underwent surgery, mainly to treat for concomitant pathologies (knee arthroscopy, ligament reconstruction). Only one patient with FOPE as the main pathology underwent diagnostic arthroscopy. In 15 of 93 patients (16.1%) no therapy was documented.

When restricted to the 52 patients with FOPE as main pathology, 80.8% of the cases received conservative treatment while one patient (1.9%) underwent diagnostic arthroscopy, and nine cases (17.3%) lacked documentation of treatment details.

The follow-up periods varied considerably due to individual patient specific factors, yet most FOPE-positive patients were discharged after conservative treatment and without FOPE-specific surgical interventions. While pain trajectories were not consistently quantified in our charts, no FOPE-related complications or growth disturbances were recorded.

## Discussion

In this large single-centre series of adolescent knees, FOPE was identified in roughly one in ten MRI patients. We found that the incidence was significantly higher in female adolescents, and severe FOPE occurred exclusively when FOPE represented the main pathology on MRI. The majority of patients with FOPE received conservative treatment, while surgical interventions were uncommon and rarely targeted the FOPE condition directly.

These data confirm FOPE as a common, mostly benign finding with distinct epidemiological characteristics and provide quantitative evidence supporting the cautious, non-invasive conservative treatment, as described in previous small series studies [3, 6–8].

Zbojniewicz and Laor’s original case series (12 patients, 15 knees) introduced FOPE as a potentially painful manifestation of physiological physeal fusion and recommended avoiding invasive diagnostics when the characteristic imaging pattern is present [3]. Subsequent studies of Beckmann and Spence and Bai et al. and Bochmann et al. and other researchers documented small groups of FOPE patients experiencing symptoms while focusing on unusual cases such as bilateral lesions, partial physeal closure and the progression of imaging findings during follow-up periods [4–6, 9].

Ueyama et al. provided the first structured follow-up of FOPE lesions and reported spontaneous resolution of symptoms within approximately two years under conservative treatment, without growth disturbances [7]. Giles et al. and, more recently, Speirs et al. further highlighted FOPE to the orthopaedic community and cautioned against over-treatment of what may often represent a physiological or self-limiting phenomenon [8, 9].

Our study complements and extends this body of evidence in several ways. To our knowledge, this is the first study to quantify FOPE incidence in a large consecutive cohort of adolescent knee MRIs (10.8% of patients). Existing FOPE reports do not provide population-based incidence [11].

While previous case studies suggested a female predominance, sample sizes were too small for statistical analysis. In our cohort, FOPE was significantly more frequent in girls (13.4% vs 7.6%), aligning with earlier physis closure and possibly higher participation in certain sports [4, 8].

The strong association between lesion severity and FOPE being the main pathology (severe lesions only in main-pathology cases; *p *= 0.005) provides quantitative support for the intuitive notion that more extensive FOPE is more likely to be clinically relevant.

Our data demonstrate that, in real-world practice—before FOPE was widely recognised—FOPE-positive adolescents were almost exclusively treated conservatively, with very low rates of surgery and no FOPE-specific operative interventions. This supports existing recommendations to avoid invasive strategies when FOPE is the only plausible explanation for pain [3, 7–9].

### Clinical implications

The main value of recognising FOPE is the prevention of misinterpretation of focal bone marrow signal alterations as a tumour, infection, or occult fractures. Correct classification of joint pathology can prevent unneeded medical procedures such as repeated imaging or invasive procedures including biopsy or arthroscopy.

Our data show that FOPE is a common finding in MRI for adolescent knee pain. Severe FOPE can be the only structural abnormality found in symptomatic knees without additional pathology. Our study group showed that identifying FOPE as a sole MRI finding may pose a diagnostic challenge for radiologists and orthopaedic surgeons, therefor, increased awareness of this condition is essential.

We recommend a practical method for handling FOPE findings on adolescent MRI scans based on current evidence and our cohort data. Evaluation should begin with a thorough clinical examination to assess knee pain and exclude other potential causes, including ligament or meniscal injuries, osteochondral lesions, and infection. Conservative management should be first-line treatment when FOPE is the only relevant MRI abnormality or clearly dominant lesion.

Invasive diagnostic procedures (biopsy, diagnostic arthroscopy) performed solely due to FOPE should be avoided unless atypical imaging features or persistent, unexplained symptoms raise concern for alternative pathology [3, 7, 8].

Although the present retrospective dataset does not include standardised pain scores, knee pain was documented in 90% of FOPE-positive patients, and FOPE frequently represented the main MRI pathology, particularly in more severe lesions. The data demonstrate that severe lesions always receive the designation of "main pathology" which supports the idea that FOPE produces symptoms in certain patients. Our findings are consistent with case-based evidence showing temporal correlation between FOPE and pain, and improvement under conservative management [3, 5, 7, 9].

The current study also quantified treatment patterns, showing that FOPE-positive patients were predominantly treated conservatively and rarely required surgery when FOPE served as the primary diagnosis.

The available data set lacks MRI follow-up imaging for most patients, therefore, we could not provide robust statistically significant MRI follow up data. Due to the retrospective analysis relying on documented data, management was focused on patients’ symptoms. Resolution of symptoms provided the end point of treatment concurrent with existing research [7, 9].

### Limitations

The study has several limitations. Its retrospective, single-centre design may limit generalisability. Clinical data (pain intensity, functional scores) were not prospectively collected, and documentation quality varied. FOPE classification and severity grading were based on expert consensus without formal inter-observer testing. We did not systematically capture long-term clinical outcomes or radiologic resolution of FOPE. Finally, our incidence estimates apply to a population already selected for MRI (i.e. symptomatic adolescents) and cannot be directly extrapolated to the general adolescent population.

## Conclusion

FOPE is a relatively common MRI finding in adolescents with knee pain, occurring in approximately one in ten symptomatic knees and significantly more often in girls. Severe FOPE lesions are strongly associated with FOPE being the main pathology, while milder lesions frequently coexist with other causes of knee pain. In this large clinical cohort, FOPE-positive patients were treated almost exclusively conservatively, with surgery rarely indicated.

Recognising FOPE as a likely benign, self-limiting manifestation of physeal maturation in many cases—but potentially symptomatic in others—can help orthopaedic surgeons and radiologists avoid unnecessary invasive diagnostics while still considering FOPE as a relevant differential diagnosis in adolescents with unexplained knee pain. Prospective studies with standardised clinical assessment, serial imaging, and formal inter-observer analysis are warranted to further clarify the natural history and clinical significance of FOPE.

## Data Availability

Identifiable data, including medical records and MRI scans, were generated during this study. These data are stored on the secure server of University Hospital Innsbruck and may be made available in a de-identified form upon reasonable request to the corresponding author, subject to applicable data protection regulations and institutional policies.
